# SARS-CoV-2 Secondary Attack Rates in Vaccinated and Unvaccinated Household Contacts during Replacement of Delta with Omicron Variant, Spain

**DOI:** 10.3201/eid2810.220494

**Published:** 2022-10

**Authors:** Israel López-Muñoz, Ariadna Torrella, Olga Pérez-Quílez, Amaia Castillo-Zuza, Elisa Martró, Antoni E. Bordoy, Verónica Saludes, Ignacio Blanco, Laura Soldevila, Oriol Estrada, Lluís Valerio, Sílvia Roure, Xavier Vallès

**Affiliations:** North Metropolitan International Health Program, Catalan Institute of Health (PROSICS), Badalona, Spain (I. López-Muñoz, A. Torrella, O. Pérez-Quílez, A. Castillo-Zuza, L. Soldevila, L. Valerio, S. Roure, X. Vallès);; Hospital Universitari Germans Trias i Pujol, Badalona (E. Martró, A.E. Bordoy, V. Saludes, I. Blanco, L. Soldevila, S. Roure);; Center for Biomedical Research in Epidemiology and Public Health (CIBEREPS), Instituto de Salud Carlos III, Madrid, Spain (E. Martró, A.E. Bordoy, V. Saludes);; Institut d’Investigació en Ciències de la Salut Germans Trias i Pujol, Badalona (E. Martró, A.E. Bordoy, V. Saludes, I. Blanco, O. Estrada, X. Vallès);; Fundació Lluita contra la Sida i les Malalties Transmissibles, Badalona (L. Soldevila, S. Roure, X. Vallès)

**Keywords:** COVID-19, respiratory infections, severe acute respiratory syndrome coronavirus 2, SARS-CoV-2, SARS, coronavirus disease, zoonoses, viruses, coronavirus, contact tracing, vaccine effectiveness, Delta variant, Omicron variant, secondary attack rate, Spain

## Abstract

We performed a prospective, cross-sectional study of household contacts of symptomatic index case-patients with SARS-CoV-2 infection during the shift from Delta- to Omicron-dominant variants in Spain. We included 466 household contacts from 227 index cases. The secondary attack rate was 58.2% (95% CI 49.1%–62.6%) during the Delta-dominant period and 80.9% (95% CI 75.0%–86.9%) during the Omicron-dominant period. During the Delta-dominant period, unvaccinated contacts had higher probability of infection than vaccinated contacts (odds ratio 5.42, 95% CI 1.6–18.6), but this effect disappeared at ≈20 weeks after vaccination. Contacts showed a higher relative risk of infection (9.16, 95% CI 3.4–25.0) in the Omicron-dominant than Delta-dominant period when vaccinated within the previous 20 weeks. Our data suggest vaccine evasion might be a cause of rapid spread of the Omicron variant. We recommend a focus on developing vaccines with long-lasting protection against severe disease, rather than only against infectivity.

The mass vaccination against SARS-CoV-2 that began at the end of 2020 reduced COVID-19-related mortality and severity in countries where substantial vaccine coverage was achieved (*1*,*2*). The vaccines also had a protective effect against the most recent variants (*3*,*4*). However, expectations that vaccines would stop community transmission of SARS-CoV-2 through herd immunity were quickly dampened by the early observation of infection and re-infection among vaccinated persons; waning vaccine effectiveness against transmission (VET) over time was observed (*1*,*3*) and confirmed in a large systematic literature review (*5*). Despite these results, protective effects of vaccination against infection among contacts have been reported (*6*). The vaccination status of index case-patients was also shown to play a role (*6*), underscoring the importance of vaccination for reducing the circulation of SARS-CoV-2. Nonetheless, the emergence of new variants of concern (VOC) with increased infectivity is an ongoing challenge for VET of currently licensed vaccines; early reports have shown a substantially lower VET for the Delta variant (B.1.617.2) compared with previous VOCs (*7*). Furthermore, rapid replacement of the Delta variant by Omicron (B.1.1.529) began in late 2021; the Omicron variant showed a transmission advantage because of its shorter generation time (S. Abbott et al., unpub. data, https://www.medrxiv.org/content/10.1101/2022.01.08.22268920v1). 

Evaluating both variant virulence and SARS-CoV-2 VET under high vaccine coverage levels has major epidemiologic, social, and policy implications. We report the results of an observational study of household contacts of SARS-CoV-2–infected index case-patients during a Delta variant–dominant period from September to December 2021 and an Omicron variant–dominant period during January 2022 in a north-metropolitan area of Barcelona, Spain. We evaluated the protective effects of vaccination status, time elapsed since vaccine administration, absolute and relative infectiousness of both variants, overall VET, and VET relative to vaccination status for index case-patients and contacts during both periods. The study was approved by the Ethics Board of the Hospital Universitari Germans Trias i Pujol, Badalona, Catalonia, Spain (reference no. PI-20-228) and conducted in accordance with the principles of the Declaration of Helsinki. Oral informed consent was obtained from all individual participants included in the study.

## Materials and Methods

### Study Population

The study population catchment area was the northern part of the greater metropolitan area of Barcelona in Catalonia, Spain. The area has ≈800,000 inhabitants and comprises a mixture of urban and semirural municipalities. During the study period, SARS-CoV-2 screening was readily available at no cost to persons with suspected COVID-19 and their contacts at health centers serving the respective primary care catchment areas. The smallest administrative area of the public healthcare system in Catalonia typically covers 15,000–25,000 inhabitants.

### Study Design

**Figure 1:**
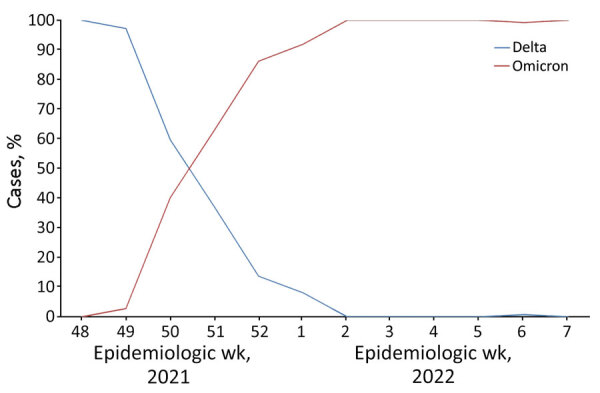
Dominance of infection with SARS-CoV-2 Delta and Omicron variants in a study of secondary attack rates in vaccinated and unvaccinated household contacts, Spain. The study population was located in the northern part of the greater metropolitan area of Barcelona, Spain. Genotyping of 1,554 samples from patients with SARS-CoV-2 infections was conducted during November 23, 2020–February 8, 2021 to identify the dominant variant infecting the population. The cutoff date between the Delta and Omicron predominance periods was December 21, 2021.

We performed a prospective cross-sectional study of household contacts of symptomatic index case-patients who had SARS-CoV-2 infection during September 21, 2021–February 7, 2022. Infection was determined in primary health centers by using either reverse transcription PCR (RT-PCR) or rapid antigen detection tests (Ag-RDT). The cutoff date between the Delta and Omicron dominant periods was December 21, 2021, which was determined on the basis of data from the epidemiologic surveillance system operating in the study area (Figure 1) (*8*). To evaluate differences between Delta and Omicron clusters, we estimated the relative risk (RR) of infection for contacts between the first tertile of the study period, when the Delta variant was clearly dominant, and the last tertile, when Omicron was clearly dominant.

Symptoms of SARS-CoV-2 infection were fever or clinical signs of upper respiratory tract infection. Index case-patients were those who first showed clinical symptoms of infection in a specific household and sought diagnosis or treatment at a primary healthcare center. The patient and COVID-19 epidemiologic surveillance system were notified after infection was confirmed. Index cases were included consecutively after notification but randomly chosen for subsequent data collection. We included only the index case-patients who provided >1 household contacts. 

**Figure 2:**
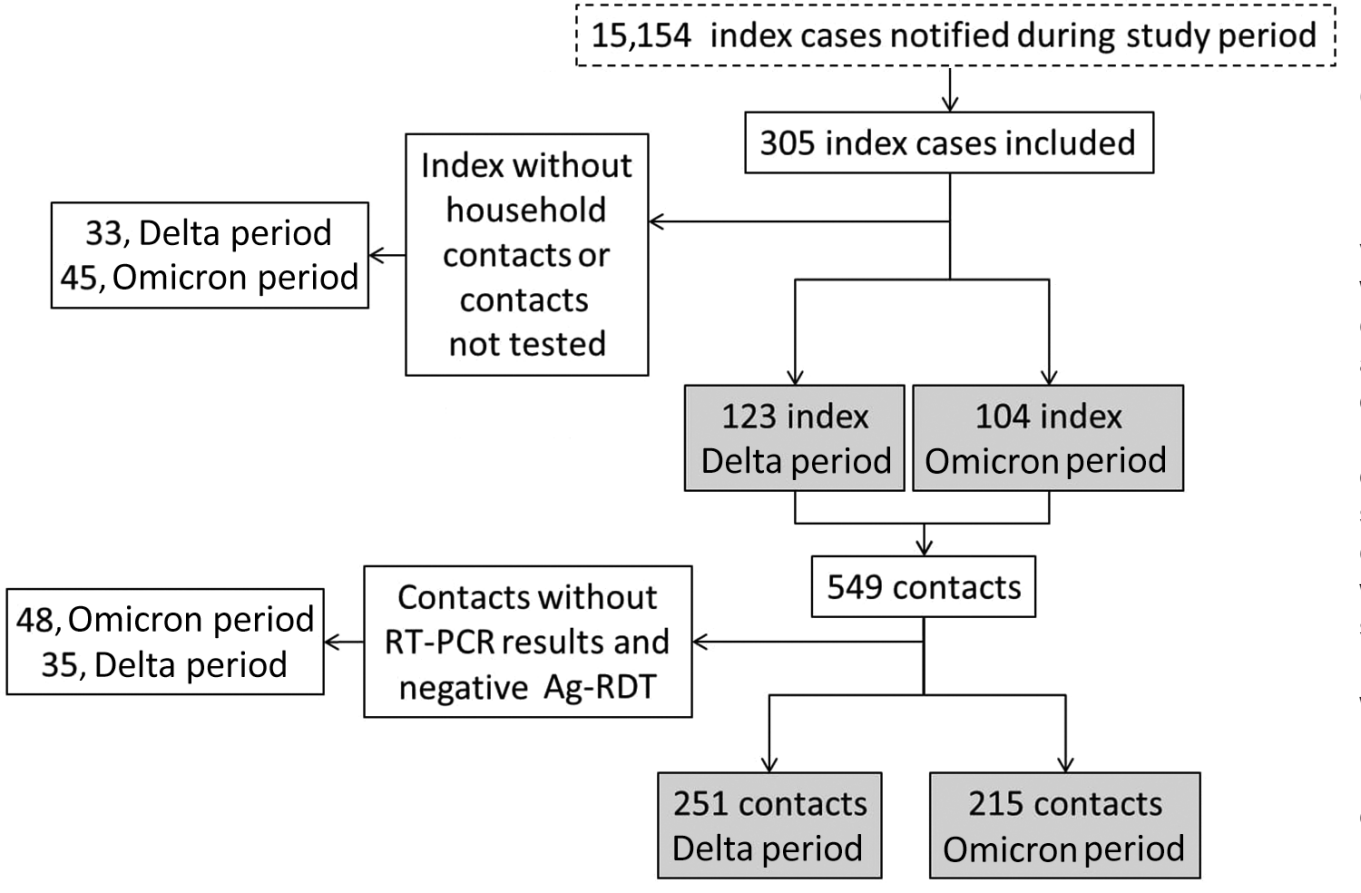
Selection process of participants in a study of SARS-CoV-2 secondary attack rates in vaccinated and unvaccinated household contacts during replacement of Delta with Omicron variant, Spain. Index case-patients were those who first showed clinical symptoms of infection in a specific household and sought diagnosis or treatment at a primary healthcare center. Contacts were defined as persons who had spent >15 min with the index case-patient in an indoor space without intervention measures, such as masks, during the 48 hours before COVID-19 diagnosis was confirmed for the index case-patient. Contacts with no RT-PCR results and negative Ag-RDT were excluded from the study. Ag-RDT, rapid antigen detection tests; RT-PCR, reverse transcription PCR.

We followed up and screened contacts according to standard procedures implemented in the study region. In brief, after confirmation of a positive case by either SARS-CoV-2–specific RT-PCR or Ag-RDT, a health officer began a systematic contact tracing study. Contacts were defined as persons who had spent >15 min with the index case-patient in an indoor space without nonpharmaceutical intervention measures during the 48 hours before COVID-19 diagnosis was confirmed for the index case-patient. This category included all housemates who were living with the index case-patient. For contacts, we performed an Ag-RDT test if the person was symptomatic at the time of the contact tracing study. We subsequently tested all contacts with a negative Ag-RDT test by RT-PCR from 3 to 7 days after the notification of the index case-patient, irrespective of the presence of symptoms. We excluded persons without available laboratory test results and those for whom RT-PCR was not performed after a negative Ag-RDT result (Figure 2). Clinical data and test results were recorded in the healthcare system’s electronic database.

### Diagnosis of SARS-CoV-2 Infection

Clinical samples collected through nasopharyngeal swabs were shipped to the referral laboratory (Microbiology Services, Metropolitan Clinical Laboratory, Hospital Universitari Germans Trias i Pujol), where they were stored at 4°C before chemical inactivation using lysis buffer. SARS-CoV-2 infection was diagnosed by using either the Novaplex SARS-CoV-2/FluA/FluB/RSV RT-PCR Assay (Seegene Inc., https://www.seegene.com) or Aptima SARS-CoV-2 assay (Hologic, https://www.hologic.com) according to the manufacturers’ instructions. Panbio Ag-RDT kits (Abbott, https://www.abbott.com) were used in situ at primary healthcare centers according to the manufacturer’s instructions.

### Data Management and Statistical Analysis

We collected data from the public health system’s electronic records and obtained additional sociodemographic data from contacts or close informants through telephone interviews. Data included RT-PCR and Ag-RDT results for contacts, presence of symptoms, background of previous COVID-19 diagnosis (defined as a previous positive SARS-CoV-2 RT-PCR test or Ag-RDT), age, sex, vaccination status against SARS-CoV-2, vaccine brand if applicable, number of vaccine doses administered (1–3), date of vaccine inoculations, and number of housemates. The vaccines licensed in our study setting were AZD1222 (ChAdOx1 nCoV-19; AstraZeneca, https://www.astrazeneca.com), BNT162b2 (Pfizer-BioNTech, https://www.pfizer.com), mRNA-1273 (Moderna, https://www.modernatx.com), and JNJ-78436735/Ad26.COV2.S (Janssen/Johnson & Johnson, https://www.jnj.com). Contacts were considered positive COVID-19 cases if either RT-PCR or Ag-RDT was positive for SARS-CoV-2 during the contact tracing period from 48 hours before to 7 days after notifying the index case-patient. For contacts, we used 2 definitions for vaccination status: vaccinated with any dose or stratified according to the number of doses received. Full vaccination was considered to be 2 or 3 doses. 

We analyzed data using Stata version 14.0 (StataCorp LLC, https://www.stata.com) and R version 4.1.2 (The R Project for Statistical Computing, https://www.r-project.org) software. For descriptive analysis, we used medians and interquartile ranges (IQRs) for continuous variables and proportions and 95% CIs for categorical variables. For univariate analysis, we used the χ^2^ test to compare categorical variables and for trends, when appropriate, and Student *t*-test for continuous variables after testing for normality (skewness and kurtosis tests) or nonparametric Fisher or Wilcoxon tests, when necessary. We performed logistic regression for multivariate analysis and estimated crude odds ratios (ORs), RRs, adjusted ORs (aORs), or adjusted RRs for study variables, including age of contacts and index-patients, sex, vaccination status, and number of housemates. We performed regression analysis to compare continuous variables, calculated crude ORs and aORs, and estimated 95% CIs and p values. We considered a p value <0.05 significant.

## Results

### Study Sample

We included 227 symptomatic index case-patients who reported a total of 466 household contacts; median number of contacts was 2 (IQR 2–3, range 1–7). The Delta-dominant period had 123 index cases and 251 contacts, and the Omicron-dominant period had 104 index cases and 215 contacts (Figure 2). The median age for the entire sample of 693 participants (index patients plus contacts) was 38.0 (IQR 15.0–49.5, range 1–91) years; 347 participants (50.1%) were female, and 511 (73.7%, 95% CI 70.3%–77.0%) were vaccinated (Appendix Table). Vaccination levels increased to 89.9% (491/546, 95% CI 87.1%–92.3%) when we excluded children <12 years of age, for whom vaccination was not implemented until mid-December 2021. Among vaccinated persons, 12.9% (66/511, 95% CI 10.1%–16.1%) were vaccinated with >1 dose of ChAdOx1-S vaccine, 3.9% (20/511, 95% CI 2.4%–6.0%) with JNJ-78436735/Ad26.COV2.S, 20.0% (102/511, 95% CI 16.6%–23.7%) with mRNA-1273, and 71.8% (367/511, 95% CI 67.7%–65.7%) with BNT162b2 (Appendix Table). Unvaccinated persons tended to be younger (median 10 [IQR 6.0–21.0] years of age) compared with those who were vaccinated (median 43.0 [IQR 27.0–53.0] years of age), mainly because of the overrepresentation of children in the unvaccinated group (p<0.001). Overall, 87.5% (447/511, 95% CI 84.3%–90.2%) of vaccinated adults had received a full vaccine course, most often 2 doses of BNT162b2 (295/511, 57.7%, 95% CI 53.3%–62.1%). The median time from the index case report date to the administration of the last vaccine dose among contacts was 20.4 (IQR 14.3–25.0, range 0.1–46) weeks. Index case-patients from the Omicron-dominant period tended to be younger (39.0 [IQR 19.3–48.0] years of age) than those from the Delta-dominant period (43.0 [IQR 25.0–55.0] years of age; p = 0.03). Contacts from the Omicron-dominant period had a higher prevalence of symptoms (57.1%, 95% CI 50.0%–64.0%) than those from the Delta-dominant period (46.4%, 95% CI 34.9%–53.0%; p = 0.03). The overall vaccination coverage was higher among persons during the Omicron-dominant period (79.3%, 95% CI 64.0%–73.6%) than during the Delta-dominant period (69.0%, 95% CI 74.4%–83.6%; p = 0.002). The secondary attack rate (SAR) was higher in contacts during the Omicron-dominant period (58.2%, 95% CI 51.8%–64.3%) than during the Delta-dominant period (80.9%, 95% CI 75.0%–86.9%; p<0.001) (Appendix Table). 

### Risk Factors for Infection among Contacts

During the Delta-dominant period, independent risk factors associated with infection were unvaccinated status (aOR 5.42, 95% CI 1.6–18.6), elapsed time since last vaccine dose (pooled aOR 1.63, 95% CI 1.1–2.4), and older age (pooled aOR 1.48, 95% CI 1.1–1.9) (Table 1). We observed a protective association between unvaccinated status of index cases and infection risk of contacts (aOR 0.30, 95% CI 0.1–0.8) in the Delta-dominant period (Table 1). We did not observe any associations between study variables and infection risk for contacts during the Omicron period (Table 2). Only 1 of 9 contacts with a previous SARS-CoV-2 infection was re-infected during the Delta-dominant period (p = 0.002), but we did not observe this effect during the Omicron-dominant period (Tables 1, 2).

**Table 1:** Crude and adjusted risk factors for infection among contacts in the Delta-dominant period in a study of SARS-CoV-2 secondary attack rates in vaccinated and unvaccinated household contacts during replacement of Delta with Omicron variant, Spain*

Variable	No. patients†	Crude OR			Adjusted OR‡	
		OR (95% CI)	p value		OR (95% CI)	p value
Vaccination status, contacts						
Vaccinated	88/167	Referent			Referent	
Unvaccinated	58/84	2.00 (1.2–3.5)	0.01		5.42 (1.6–18.6)	0.007
1 dose	8/17	Referent			Referent	
2 doses	77/146	1.25 (0.5–3.4)	0.7		1.26 (0.4–3.8)	0.7
3 doses	3/4	3.37 (0.3–39.3)	0.3		2.12 (0.2–27.2)	0.5
Time since vaccination, wk						
1–13	11/31	Referent			Referent	
14–20	20/50	1.21 (0.5–3.1)	0.7		0.98 (0.4–2.7)	0.9
21–25	32/54	2.64 (1.1–6.6)	0.04		1.74 (0.6–5.0)	0.3
>25	25/31	7.58 (2.4–24.1)	0.001		4.17 (1.1–15.3)	0.03
Missing data	0/1					
Pooled		1.96 (1.4–2.8)	<0.001		1.63 (1.1–2.4)	0.01
Age of contacts, y						
0–12	45/70	Referent			Referent	
13–18	7/18	0.35 (0.1–1.0)	0.06		1.50 (0.3–6.8)	0.6
19–35	14/32	0.43 (0.2–1.0)	0.05		1.62 (0.4–6.4)	0.5
36–45	24/46	0.61 (0.3–1.3)	0.2		2.68 (0.7–10.1)	0.1
>45	53/81	1.05 (0.5–2.1)	0.9		4.45 (1.1–18.3)	0.04
Missing data	3/4	1.7 (0.2–16.9)	0.5			
Pooled		1.03 (0.9–1.2)	0.7		1.48 (1.1–1.9)	0.003
Vaccination status, index patients						
Vaccinated	114/180	Referent			Referent	
Unvaccinated	32/71	0.48 (0.3–0.8)	0.009		0.30 (0.1–0.8)	0.02
Age of index patients, y						
0–12	21/48	Referent			Referent	
13–18	4/6	2.57 (0.4–15.4)	0.3		0.54 (0.1–4.6)	0.6
19–35	20/38	1.43 (0.6–3.4)	0.4		0.40 (0.1–1.4)	0.1
36–45	46/69	2.57 (1.2–5.5)	0.02		0.71 (0.2–2.3)	0.6
>45	55/90	2.02 (1.0–4.1)	0.05		0.57 (0.2–1.9)	0.4
Pooled		1.20 (1.0–1.4)	0.03		0.94 (0.7–1.2)	0.6
Number of housemates						
<2	70/104	Referent			Referent	
>2	58/116	0.52 (0.3–0.9)	0.01		0.63 (0.3–1.2)	0.1
Sex						
M	79/132	Referent			Referent	
F	67/118	1.13 (0.7–1.9)	0.6		1.03 (0.6–1.8)	0.9
Missing data	0/1					

*OR, odds ratio. †Values are number infected/total number of patients in each strata. ‡Adjusted analysis only included participants who had all data available.

**Table 2:** Crude and adjusted risk factors for infection among contacts in the Omicron-dominant period in a study of SARS-CoV-2 secondary attack rates in vaccinated and unvaccinated household contacts during replacement of Delta with Omicron variant, Spain*

Variable	No. patients†	Crude OR			Adjusted OR‡	
		OR (95% CI)	p value		OR (95% CI)	p value
Vaccination status, contacts						
Vaccinated	135/170	Referent			Referent	
Unvaccinated	39/45	1.69 (0.7–4.3)	0.3		1.86 (0.6–6.2)	0.3
1 dose	25/29	Referent			Referent	
2 doses	90/113	0.63 (0.2–2.0)	0.4		0.75 (0.2–2.9)	0.7
3 doses	19/27	0.38 (0.1–1.5)	0.2		0.36 (0.1–1.9)	0.2
Missing data	1/1					
Time since vaccination, wk						
1–13	36/48	Referent			Referent	
14–20	28/35	1.33 (0.5–3.8)	0.6		2.17 (0.5–9.3)	0.3
21–25	34/41	1.62 (0.6–4.6)	0.4		2.41 (0.7–7.8)	0.1
>25	36/45	1.33 (0.5–3.6)	0.6		1.91 (0.6–5.7)	0.2
Missing data	1/1					
Pooled		1.12 (0.8–1.5)	0.5		1.26 (0.9–1.8)	0.2
Age of contacts, y						
0–12	36/42	Referent			Referent	
13/18	22/27	0.73 (0.2–2.7)	0.6		0.99 (0.2–4.4)	0.9
19/35	38/47	0.70 (0.2–2.2)	0.5		0.94 (0.2–3.8)	0.9
36/45	33/42	0.61 (0.2–1.9)	0.4		0.83 (0.2–3.3)	0.8
>45	45/57	0.63 (0.2–1.8)	0.4		0.82 (0.2–3.2)	0.8
Pooled		0.90 (0.7–1.1)	0.4		0.94 (0.7–1.2)	0.6
Vaccination status, index patients						
Vaccinated	134/161	Referent			Referent	
Unvaccinated	40/54	0.56 (0.3–1.2)	0.1		0.98 (0.2–3.9)	0.9
Age of index patients, y						
0–12	36/50	Referent			Referent	
13–18	13/15	2.53 (0.5–12.7)	0.3		2.79 (0.4–20.4)	0.3
19–35	35/44	1.51 (0.6–3.9)	0.4		1.47 (0.4–5.8)	0.6
36–45	51/59	2.48 (0.9–6.5)	0.07		2.27 (0.5–10.5)	0.3
>45	39/47	1.90 (0.7–5.0)	0.2		1.84 (0.4–8.8)	0.4
Pooled		1.20 (0.9–1.5)	0.1		1.09 (0.8–1.5)	0.6
Number of housemates						
<2	60/70	Referent			Referent	
>2	114/145	0.61 (0.3–1.3)	0.2		0.62 (0.3–1.4)	0.3
Sex						
M	93/115	Referent			Referent	
F	81/100	1.13 (0.7–1.9)	0.6		0.97 (0.5–2.0)	0.9

*OR, odds ratio. †Values are number infected/total number of patients in each strata. ‡Adjusted analysis only included participants who had all data available.

### Infection Risk for Contacts during Delta- versus Omicron-Dominant Periods

The adjusted RR of infection among contacts was 3.87-fold (95% CI 2.4–6.2-fold) higher during the Omicron-dominant period than the Delta-dominant period. Analysis of RR of infection was restricted to the first and last tertiles of the study period for vaccinated and unvaccinated contacts and index case-patients (Table 3). Contacts during the Omicron-dominant period showed a higher RR of infection than those in the Delta-dominant period for all strata studied. However, this effect was more prominent among contacts who were vaccinated <20 weeks before contact with the index-case patient (RR 9.16, 95% CI 3.4–25.0) compared with those who were vaccinated >20 weeks before contact with the index-case patient (RR 2.91, 95% CI 0.8-10.2).

**Table 3:** Risk of infection among contacts relative to vaccination status in a study of SARS-CoV-2 secondary attack rates in vaccinated and unvaccinated household contacts during replacement of Delta with Omicron variant, Spain*

Variable	Delta-dominant period			Omicron-dominant period		RR§ (95% CI)	p value
	Patients†	p value‡		Patients†	p value‡		
Vaccination status, contacts							
Vaccinated	50/101 (49.5)	0.1		112/124 (90.3)	0.3	6.48 (3.0–13.8)	<0.001
Unvaccinated	35/56 (62.5)			28/29 (96.6)		10.4 (1.2–82.5)	0.03
Vaccinated, <20 wk	23/62 (37.1)	0.002		59/65 (90.8)	0.8	9.16 (3.4–25.0)	<0.001
Vaccinated, >20 wk	27/39 (69.3)			52/58 (89.7)		2.91 (0.8–10.2)	0.1
Vaccination status, index							
Vaccinated	67/112 (59.8)	0.02		108/120 (90.0)	0.2	3.99 (2.0–8.1)	<0.001
Unvaccinated	18/45 (40.0)			32/33 (97.0)		43.5 (5.1–369.9)	0.001

*RR, relative risk †Values are no. infected/total no. (%) patients in each strata. ‡p values for differences between vaccinated and unvaccinated groups. §RR between Omicron vs. Delta variant, adjusted by age of contact.

**Figure 3:**
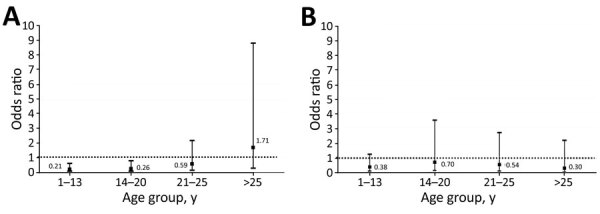
Association between time elapsed since the last vaccination and infection risk in a study of SARS-CoV-2 secondary attack rates in vaccinated and unvaccinated household contacts during replacement of Delta with Omicron variant, Spain. Odds ratios for infection risk of contacts were calculated for each age group in the Delta-dominant period (A) and Omicron-dominant period (B). Data were stratified according to interquartile range distribution of vaccinated contacts and number of weeks that elapsed since their last vaccination dose. Unvaccinated contacts were used as the control group for comparison. Dashed lines indicate the no-association threshold (odds ratio = 1).

To explore the time lag effect since vaccine administration, we stratified the group of vaccinated contacts according to the IQR and number of weeks that elapsed since their last vaccination dose and compared each group with unvaccinated contacts. We found a protective effect for VET in vaccinated compared with unvaccinated persons in the first 2 strata that were closer to the vaccination date during the Delta-dominant period after adjusting for age (OR 0.21, 95% CI 0.1–0.7, p = 0.007, and OR 0.26, 95% CI 0.1–0.9, p = 0.03), but not for the Omicron-dominant period (Figure 3). This protective effect during the Delta-dominant period disappeared in the upper IQR strata (>20 weeks) (Figure 3).

We observed no significant differences for age of contacts in all time lag strata. We reassessed these results using only data for contacts who had received 2 or 3 vaccine doses and observed similar results. We plotted age against time elapsed since vaccination after stratifying according to the infection status of contacts and used linear regression analysis to visualize the effects of age on infection risk during the 2 study periods (Appendix Figure 1). 

## Discussion

Our results show a high SAR among household contacts for both the Delta-dominant (58.2%) and Omicron-dominant (80.9%) periods; we found a 2- to 6-fold higher risk of infection for household contacts of symptomatic index case-patients during the Omicron-dominant period. SARs in our study were higher than that observed in a previous study conducted in the same geographic area in 2020, which showed a secondary infection rate of 48.3% when the ancestral SARS-CoV-2 strain responsible for the first infection wave was predominant (*9*). This previous study (*9*) classified hospitalized persons as index case-patients, and the mapping of those index cases demonstrated clear clustering in geographic areas with lower socio-economic status. In our study, we did not observe geographic aggregation of index case-patients and contacts in either variant-dominant period, which might indicate intrinsically higher infectious capacity and community penetrance of the Delta and Omicron variants compared with previous variants and explain their markedly high SAR. Despite high vaccine coverage, infection during the Delta- and Omicron-dominant periods occurred regardless of other socio-economic factors previously observed, such as the number of housemates (*10*). Furthermore, for the Delta variant, the SAR observed in our study was higher than those reported among household contacts in England (25%) (*11*), Denmark (21%) (F.P. Lyngse et al., unpub. data, https://www.medrxiv.org/content/10.1101/2021.12.27.21268278v1), Japan (25.2%) (*12*), Northern Spain (24%) (*13*), and those published in a systematic review and meta-analysis (22.5%) (*14*) but was similar to the 43.1% SAR reported in South Korea (*15*). For the Omicron variant, the SAR in our study was higher than those reported in Denmark (31%) (F.P. Lyngse et al., unpub. data), Japan (31.8%) (*12*), and the United States (52.7%) (*16*), and an overall rate of 42.7% (*14*). In our study, we included only symptomatic index case-patients. Symptomatic SARS-CoV-2–infected patients might be more efficient transmitters of the virus (*17*) because they maintain higher viral loads for a longer period (*18*) and might spread the infection more efficiently through sneezing or coughing (*19*). The higher SARs in our study might also reflect a low level of compliance with isolation measures among index case-patients within households or different testing and inclusion strategies. Nevertheless, our results indicate that the Omicron variant and, to a lesser extent, the Delta variant have an extremely high transmission capacity among close contacts, irrespective of vaccination status and other co-factors.

Vaccine evasion might be a contributor to the higher transmissibility of the Omicron variant in areas with high vaccine coverage (F.P. Lyngse et al., unpub. data). This conclusion is supported by the substantial RR of SARS-CoV-2 infection in contacts during the Omicron-dominant period who were vaccinated within 20 weeks before infection by the index case-patients but not in contacts vaccinated at >20 weeks before infection (Table 3). These observations are consistent with the reduction of neutralizing antibodies against Omicron observed in experimental studies (*20*,*21*) and a notable duration of infectious shedding of the Omicron virus in vaccinated persons (*22*). The protective effect of vaccination against SARS-CoV-2 infection in the Delta-dominant period was consistent with previous reports on the Delta VOC, which had similar but limited results (*6*,*21*,*23*–*26*). However, we could not ascertain if the booster dose was effective for reducing transmission, likely because of the low sample size (only 46 contacts had booster vaccines administered during the Delta-dominant period). The protective effect against the Delta variant diminished as the time since vaccination increased, which has also been previously reported (*5*,*24*,*26*). Our estimates suggest a nonlinear trend for reduction of vaccine protection, culminating at ≈20 weeks after vaccination. The underlying mechanisms might include a rapid decline of vaccine-induced peak IgA, which is a mucosal antibody with more potent neutralizing activity than IgG (*27*) against the spike protein (*28*). A substantial reduction of IgA was also observed 3 months following natural infection (*29*), which is compatible with our results, considering that IgA remains longer in mucosal fluids than serum (*29*).

We cannot conclude that the vaccination status of the index case-patients provided protection against infection for their contacts. However, we suggest that a complex relationship exists between vaccination status, immunity, and age. Children, who have shown a lower susceptibility to SARS-CoV-2 infection (*30*), might have a lower ability to transmit the infection (*31*–*33*) and tended to be unvaccinated in our sample. However, older persons tend to have better vaccine coverage but lose vaccine-induced immunity more rapidly than younger persons (*34*,*35*) and develop symptoms (*36*). Finally, vaccinated persons might have a lower inclination to practice social distancing than unvaccinated persons (*37*). Overall, these factors might explain the protective association against infection between unvaccinated index case-patients and their contacts during the Delta-dominant period. These interactions could have implications for vaccination strategy and deserve further examination; however, they might have had little or no effect during the Omicron-dominant period. Risk factors related to infection with the Omicron variant might only be ascertained with a larger sample size of Omicron-infected households. A recent large cohort study conducted in Spain found that booster mRNA vaccine doses were moderately effective in preventing infection with the SARS-CoV-2 Omicron variant for >1 month after administration, after which protection rapidly diminished compared with the protection observed against the Delta variant (*38*).

Our study’s first limitation is that we relied on the assumption that the classification of 2 periods on the basis of molecular epidemiologic surveillance of SARS-CoV-2 variants was an acceptable proxy to compare the epidemiologic behavior of the Omicron and Delta variants. However, a misclassification of the Delta and Omicron variant clusters might have occurred, especially during the middle tertile of our study period, when variants within the population overlapped. We overcame this limitation by restricting data analysis to the first and last tertiles (Table 3); however, we used all data for the remaining analyses to maintain statistical power of the study. Second, we could not confirm which persons were the true index case-patients and, therefore, some misclassification of index cases versus contacts may have occurred. In this regard, the effects of the variables studied, such as index and contact vaccination effects, might have been diluted in the study. Third, we cannot exclude the possibility that, in some households, contacts were not infected by the same index case-patient or were infected elsewhere in the community, which might again dilute the factors associated with contacts. Finally, the percentages of infected contacts in the excluded group (14% of contacts during the Delta-dominant period and 22% during the Omicron-dominant period) were lower than those for the cohort included in the study, which might have skewed the results by increasing the estimated SAR during both periods. Ultimately, full confirmation of our findings will require a longitudinal study that includes a long-term follow-up of participants and household-level genotyping results.

Our results underscore the need for continuous community-based surveillance studies to characterize the epidemiologic phenotypes of SARS-CoV-2 variants in vaccine-covered populations, especially considering the emergence of new variants, such as Omicron subvariants BA.4 and BA.5 (Appendix Figure 2). Given the increased infectiousness of the Omicron variant compared with previous VOCs, we should focus on developing vaccines with long-lasting protection against severe disease rather than only infectivity. Sustained public health measures focused on the most vulnerable populations, such as the consistent use of masks in public settings to limit infection of SARS-CoV-2, should remain a cornerstone of pandemic management. The results from this study could help healthcare policy makers formulate effective prevention policies for newly emerging VOCs.

Appendix Additional information for SARS-CoV-2 secondary attack rates in vaccinated and unvaccinated household contacts during replacement of Delta with Omicron variant, Spain.
