# Supplementary material for: SARS-CoV-2 Secondary Attack Rates in Vaccinated and Unvaccinated Household Contacts during Replacement of Delta with Omicron Variant, Spain
Source: Emerg Infect Dis. 2022 Oct;28(10):1999–2008. doi: 10.3201/eid2810.220494 (PMC9514368; doi:10.3201/eid2810.220494)
Supplement: Appendix — Additional information for SARS-CoV-2 secondary attack rates in vaccinated and unvaccinated household contacts during replacement of Delta with Omicron variant, Spain. [file 22-0494-Techapp-s1.pdf]

Article DOI: https://doi.org/10.3201/eid2810.220494

SARS-CoV-2 Secondary Attack Rates in Vaccinated and Unvaccinated Household Contacts during Replacement of Delta with Omicron Variant, Spain

Appendix

Appendix Table. Characteristics of participants in a study of SARS-CoV-2 secondary attack rates in vaccinated and unvaccinated household contacts during replacement of Delta with Omicron variant, Spain*

		Delta-dominant period			Omicron-dominant period		
Variables†	No. participants	Index cases	Contacts	p value	Index cases	Contacts	p value
Total	693	123	251		104	215	
Age group, y	38.0 (15.0–49.5)	43.0 (25.0–55.0)	37.0 (11.0–49.0)	0.003	39.0 (19.3–48.0)	31.0 (15.0–48.0)	0.1
0–12	147 (21.3)	18 (14.6)	70 (28.4)		17 (16.4)	42 (19.5)	
13–18	54 (7.8)	3 (2.4)	18 (7.3)		6 (5.8)	27 (12.6)	
19–35	123 (17.9)	22 (17.9)	32 (13.0)	0.007	22 (21.2)	47 (21.9)	0.2‡
36–45	148 (21.5)	30 (24.4)	46 (18.6)		30 (28.9)	42 (19.5)	
>45	217 (31.5)	50 (40.7)	81 (32.8)		29 (27.9)	57 (26.5)	
Missing data§	4 (0.6)	—	4 (1.6)		—	—	
Sex							
M	346 (49.9)	58 (47.1)	133 (52.8)	0.3	40 (38.5)	115 (53.5)	0.01
F	347 (50.1)	65 (52.9)	118 (47.2)		64 (61.5)	100 (46.5)	
Missing data	1 (0.1)	—	1 (0.4)		—	—	
Median no. housemates	2 (2–3)	2 (1–3)	—	—	2 (2–3)	—	—
Vaccination status							
Vaccinated (all vaccines)	511 (73.7)	91 (74.0)	167 (66.5)	0.1	83 (79.8)	170 (79.1)	0.9
AstraZeneca vaccine	66 (12.9)	18 (19.8)	22 (13.2)	0.2	11 (13.3)	15 (8.8)	0.3
Janssen vaccine	20 (3.9)	4 (4.4)	5 (3.0)	0.7	3 (3.6)	8 (4.7)	0.7
Moderna vaccine	102 (20.0)	7 (7.7)	14 (8.4)	0.8	26 (31.3)	55 (32.4)	0.9
Pfizer vaccine	367 (71.8)	63 (69.2)	127 (76.1)	0.2	54 (65.1)	123 (72.4)	0.2
Number of doses							
1 dose¶	63 (12.4)	9 (9.9)	17 (10.2)	0.9	8 (9.6)	29 (17.2)	0.1
2 doses#	401 (78.6)	77 (84.6)	146 (87.4)	0.5	65 (78.3)	113 (66.9)	0.06
3 doses††	46 (9.0)	5 (5.5)	4 (2.4)	0.2	10 (12.1)	27 (16.0)	0.4
Missing data	1 (0.2)	—	—		—	1 (0.6)	

Variables†	No. participants	Delta-dominant period		p value	Omicron-dominant period		p value
		Index cases	Contacts		Index cases	Contacts	
Median no. wk from last dose	20.4 (14.3–25.0)	22.9 (16.8–27.0)	25.1 (10.8–25.7)	0.07	20.1 (15.1–24.4)	20.0 (14.3–23.9)	0.5
Symptomatic	225 (51.4)	—	109 (46.4)	—	—	116 (57.1)	—
Missing data	28 (4.0)	—	16 (6.4)	—	—	12 (5.6)	
Previous infection	38 (7.8)	0	9 (3.6)	0.06	12 (11.5)	17 (7.9)	0.3
Missing data	208 (30.0)	71 (57.7)	129 (51.4)		1 (1.0)	7 (3.3)	
SARS-CoV-2 infection among contacts	320 (68.7)	—	146 (58.2)	—	—	174 (80.9)	—

*AstraZeneca, https://www.astrazeneca.com; Janssen, https://www.jnj.com; Moderna, https://www.modernatx.com; Pfizer-BioNtech, https://www.pfizer.com.
†Values are median (interquartile range) or no. (%).
‡p value for trend.
§Patients with missing data were not included in analysis.
¶AstraZeneca, 4/63 (6.4%); Janssen, 15/63 (23.8%); Moderna, 11/63 (17.5%); Pfizer, 33/63 (52.4%).
#AstraZeneca ×2, 49/401 (12.2%); AstraZeneca + Pfizer, 4/401 (1.0%); Janssen + Moderna, 5/401 (1.3%); Moderna ×2,48/401 (12.0%); Pfizer ×2, 295/401 (73.6%).
††AstraZeneca ×2 + Moderna, 8/46 (17.4%); AstraZeneca ×2 + Pfizer, 1/46 (2.2%); Moderna ×3, 3/46 (6.5%); Pfizer ×2 + Moderna, 27/46 (58.7%); Pfizer ×3, 7/46 (15.2%).

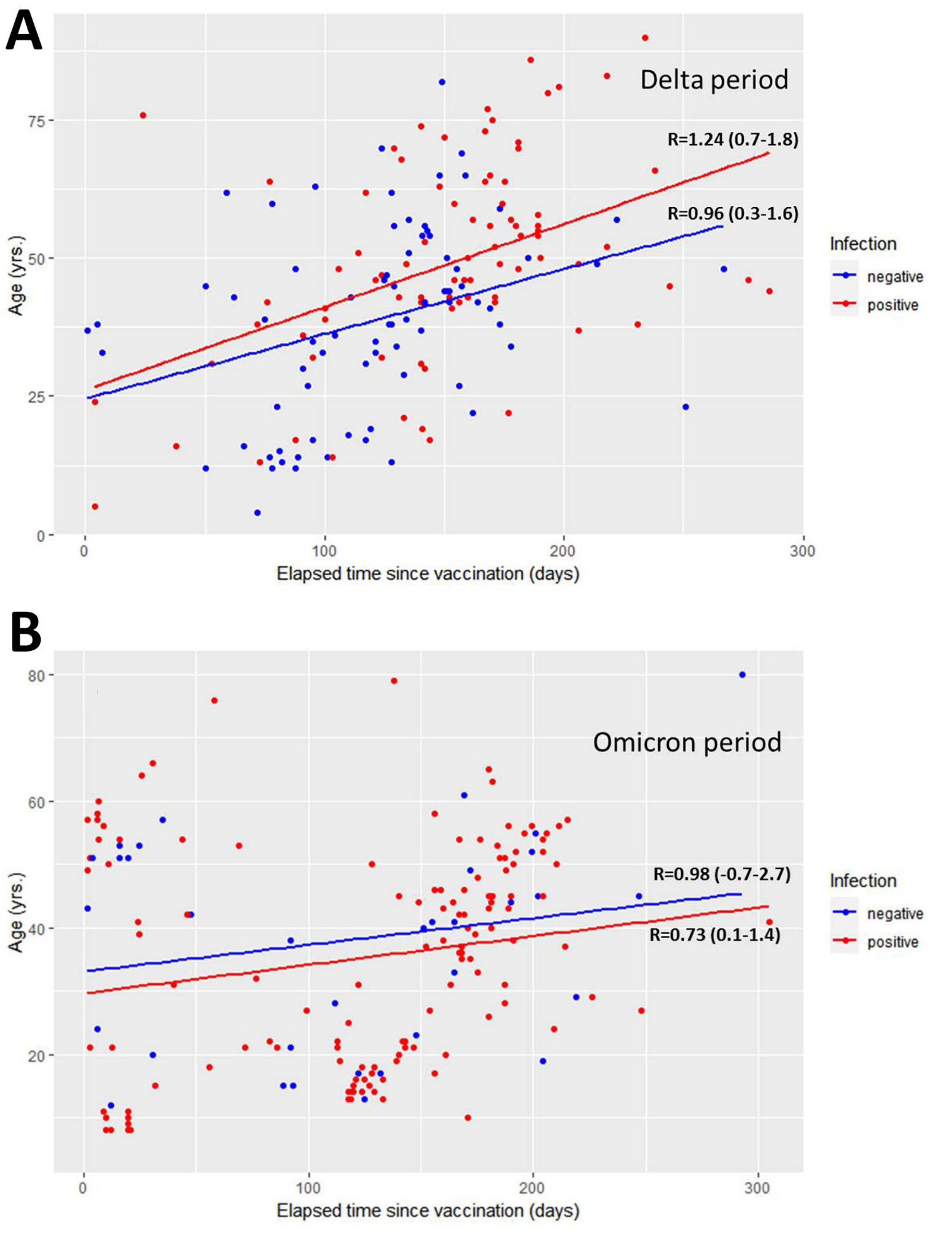

Appendix Figure 1. Effects of age on infection risk in a study of SARS-CoV-2 secondary attack rates in vaccinated and unvaccinated household contacts during replacement of Delta with Omicron variant, Spain. To visualize the effect of age on infection risk, we conducted linear regression analysis of age against time elapsed since the last vaccination during the A) Delta-dominant period and B) Omicron-dominant period after stratifying data by contact infection status. No significant differences were observed between infected and uninfected contacts in either period. R, regression coefficient (CI).

Graphical abstract

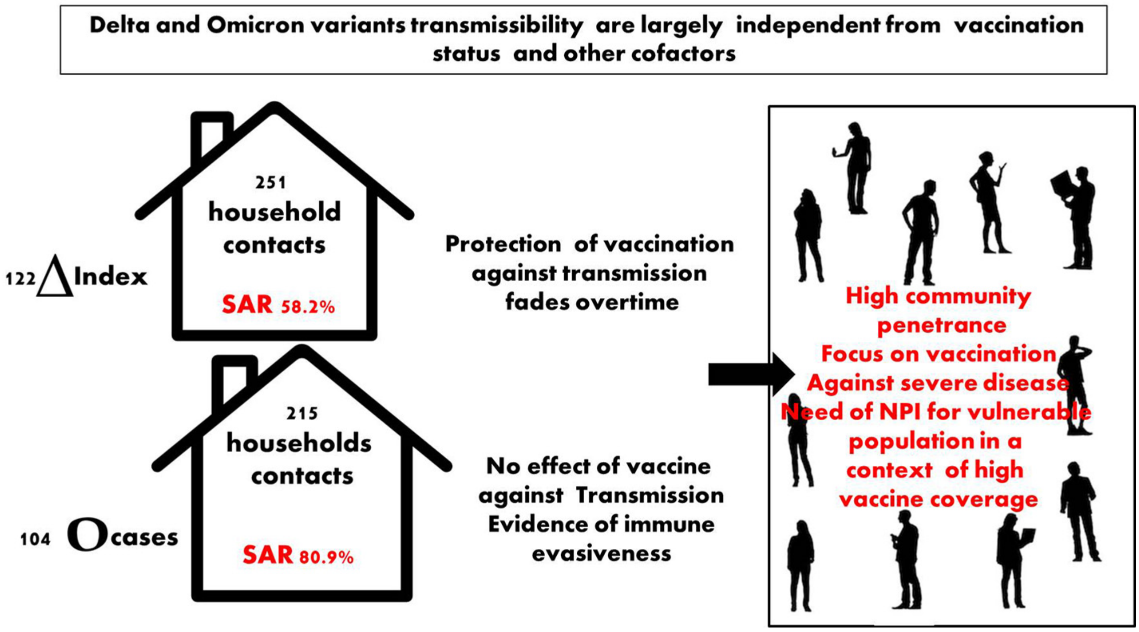

Appendix Figure 2. Graphical abstract showing SARS-CoV-2 secondary attack rates in vaccinated and unvaccinated household contacts during replacement of Delta with Omicron variant, Spain. Index case-patients were those who first showed clinical symptoms of infection in a specific household and sought diagnosis or treatment at a primary healthcare center. Contacts were defined as persons who had spent more than 15 min with the index case-patient in an indoor space without intervention measures, such as masks, during the 48 h before COVID-19 diagnosis was confirmed for the index case-patient. Vaccination had a greater protective effect against SARS-CoV-2 infection in the Delta-dominant period than Omicron period. However, protection diminished over time. In the Omicron-dominant period, vaccination had little protective effect for contacts, which was, in part, likely because of immune evasion. NPI, nonpharmaceutical intervention; SAR, secondary attack rate.
